# Atmospheric-Pressure Cold Plasma Induces Transcriptional Changes in *Ex Vivo* Human Corneas

**DOI:** 10.1371/journal.pone.0133173

**Published:** 2015-07-23

**Authors:** Umberto Rosani, Elena Tarricone, Paola Venier, Paola Brun, Velika Deligianni, Matteo Zuin, Emilio Martines, Andrea Leonardi, Paola Brun

**Affiliations:** 1 Department of Biology, University of Padova, Padova, Italy; 2 Department of Molecular Medicine, Histology Unit, University of Padova, Padova, Italy; 3 Department of Molecular Medicine, Microbiology Unit, University of Padova, Padova, Italy; 4 Department of Ophthalmology, S. Antonio Hospital, Padova, Italy; 5 Consorzio RFX, Padova, Italy; 6 Department of Neuroscience, Ophthalmology Unit, University of Padova, Padova, Italy; University Paul Sabatier, FRANCE

## Abstract

**Background:**

Atmospheric pressure cold plasma (APCP) might be considered a novel tool for tissue disinfection in medicine since the active chemical species produced by low plasma doses, generated by ionizing helium gas in air, induces reactive oxygen species (ROS) that kill microorganisms without substantially affecting human cells.

**Objectives:**

In this study, we evaluated morphological and functional changes in human corneas exposed for 2 minutes (min) to APCP and tested if the antioxidant n-acetyl l-cysteine (NAC) was able to inhibit or prevent damage and cell death.

**Results:**

Immunohistochemistry and western blotting analyses of corneal tissues collected at 6 hours (h) post-APCP treatment demonstrated no morphological tissue changes, but a transient increased expression of OGG1 glycosylase that returned to control levels in 24 h. Transcriptome sequencing and quantitative real time PCR performed on different corneas revealed in the treated corneas many differentially expressed genes: namely, 256 and 304 genes showing expression changes greater than ± 2 folds in the absence and presence of NAC, respectively. At 6 h post-treatment, the most over-expressed gene categories suggested an active or enhanced cell functioning, with only a minority of genes specifically concerning oxidative DNA damage and repair showing slight over-expression values (<2 folds). Moreover, time-related expression analysis of eight genes up-regulated in the APCP-treated corneas overall demonstrated the return to control expression levels after 24 h.

**Conclusions:**

These findings of transient oxidative stress accompanied by wide-range transcriptome adjustments support the further development of APCP as an ocular disinfectant.

## Introduction

The cornea is highly resistant to microbial invasion because of the integrity of the ocular surface and the production of various antimicrobial peptides and proteins in the tear film [1]. However, once the tear film and the epithelial integrity of the ocular surface are breached, pathogens may invade the corneal tissues, leading to microbial infections commonly termed infectious keratitis. These can result in permanent loss of vision if not rapidly and adequately treated [2]. Following corneal infection and disruption of the ocular surface, wound healing requires a complex interplay between humoral factors: cytokines, growth factors and neuropeptides, and cells such as nerve cells, stem cells, keratocytes, myofibroblasts, and dendritic cells [3–4].

Topical anti-infective drugs are normally used for the treatment of ocular infection but the increasing emergence of bacterial resistance can impact significantly the effectiveness of pharmacological therapy [5]. For this reason, new anti-infective therapeutic strategies are needed that can be efficacious in the short-term regardless of infection type. Cold plasmas obtained at atmospheric pressure are increasingly used in tissue disinfection and a new field of research called plasma medicine has developed [6–12]. Disinfection with cold plasmas might reduce the use of long-acting antibiotics in the control of superficial infections such as those of human skin, eye, and mucosal tissues exposed to the external environment.

The antibacterial effects of low temperature plasmas have been well demonstrated and depend on all products generated by plasmas, mainly by the activity of reactive oxygen species or ROS [13–14]. Antibacterial effects are also influenced by other parameters such as the gap between the plasma source and the samples, the time of exposure, the gas mixture and the plasma power applied. We previously used cold plasma (APCP) generated by helium/air ionization in a prototype device to treat different microorganisms, type of cells and tissues [15–16] at different times of exposure and different distances from the samples. We found that a 2-minute (min) flow of atmospheric pressure generates an intracellular ROS level that peaks at a 1.5 mm distance from the plasma source and exerts substantial antimicrobial effects without significant damage to cells and tissues. Moreover, the number of apoptotic nuclei in the treated corneal tissues was similar to that in control tissues.

In our device, the plasma is formed between two grids acting as electrodes. Tissues are exposed to the so-called afterglow, the chemical-enriched helium flow. Compared to other cold plasma devices proposed for biomedical applications [17], the charged species generated in our device do not reach the biological sample since they recombine very quickly, as demonstrated by the absence of light emission outside the external grid. We previously demonstrated that the UV radiation component of this APCP did not induce thymine dimers in cells and tissues treated up to 5 min; however, exposure to APCP caused a transient formation of 8-hydroxydeoxyguanosine (8-OHdG), accompanied by a temporary expression of OGG1 glycosylase, an enzyme involved in the repair of the pro-mutagenic oxidation product 8-OHdG [18,15].

The new histochemical and molecular data presented in this study aim to clarify whether the exposure of *ex vivo* human corneas to 2-min of APCP causes molecular changes to cells and tissues. We analysed whole transcriptome changes at 6 h after the exposure of corneas for 2 min to APCP in the absence or presence of the antioxidant, N-acetyl L-cysteine (NAC). Selected genes that emerged as differentially expressed after exposure to APCP or relevant to corneal pathology were further investigated by immunohistochemistry and quantitative PCR (qPCR).

## Materials and Methods

### Plasma device

Atmospheric pressure cold plasma (APCP) was produced by a device built at the Consorzio RFX in Padua, Italy. The plasma source uses a grid pair driven by a radiofrequency voltage difference of approximately 1 kV, ionizing a flow of helium gas that partially mixes with ambient air and has been described elsewhere [18,15]. Briefly, the plasma source consists of two co-axial tubes closed at the end by two parallel grids positioned at a distance of 1 mm. The outer grid is grounded, as is the whole external structure of the source, so that no electric field is present outside of the space enclosed by the two grids, where the plasma is formed. Despite the high voltage value, the current flowing in the plasma is so low that the dissipated power is below 1 W. The chosen operational frequency is 4.8 MHz.

Analysis of radiation emitted by the plasma was performed by mini-spectrometry using a Hamamatsu C10082CA integrated with a back-thinned type linear CCD image sensor (2048 pixels). This provides measurements ranging from 200 to 830 nm in a single spectrum. A quartz optic fiber with a 1 mm diameter core, 100 cm long, matched to the spectrometer, was set in front of the plasma source at a distance of 1,5 mm. The spectrum of APCP radiation obtained with a helium flow of 1.5 liters min^-1^ at room pressure displays the presence of the N_2_ molecule and high concentrations of OH radicals (309 nm) at room temperature, with most of the lines concentrated in the UV region, although this APCP is not a very effective source of high-energy UV emission [15].

### Human corneas

Eleven human corneas (HC) from healthy donors 64–72 years of age were purchased at the Veneto Eye Bank Foundation (Mestre, Italy). Written informed consent from donor relatives was obtained for the use tissues for research purposes, in agreement to the Declaration of Helsinki. The study was approved by the local ethic committee of Azienda Ospedaliera-University of Padua. Corneas were maintained in humid chambers at 37°C in D-MEM culture medium.

To evaluate transcriptome changes after exposure to APCP, sequencing libraries were prepared from nine corneas, one or two corneas per biological replicate, depending on the specimen availability when destined to multiple measures (Table 1). One cornea pair was used as an untreated control (HC1A-HC1B), to compare the tissue response to APCP treatment in the presence or absence of NAC (HC5 vs. HC3 vs.; HC6 vs. HC4 vs., respectively). Two additional corneas were used to evaluate the expression of specific genes at selected time points.

**Table 1:** Corneal samples used and RNA sequencing data report.

Sample ID	Treatment	% total reads	Mapped reads
HC1 (A, B)	control	18.9	62'848'375
HC2 (A)	control	17.9	59'615'324
HC3 (A, B)	2' APCP	16.5	54'957'538
HC4 (A)	2' APCP	16.9	56'173'853
HC5 (A, B)	2' APCP (+NAC)	15.2	50'842'261
HC6 (A)	2' APCP (+NAC)	14.7	49'112'159

Individual (HC2, HC4, HC6) or pooled (HC1, HC3, HC5) human corneas untreated or treated for 2 min with APCP in the absence or presence of N-acetyl cysteine (NAC) were subjected to RNA sequencing. HC1A-HC1B, HC3A-HC5A, HC3B-HC5B, HC4A-HC6A are cornea pair from the same donor Percentage of obtained Illumina reads on total and mapped reads per sample are shown.

#### Exposure of e*x vivo* human corneas to APCP

Human corneas were exposed to APCP for 2 min from a height of 1.5 mm, at helium flow of 1.75 l/min in DMEM medium. Untreated controls and APCP-treated corneas were then re-incubated with fresh medium for 3, 6 and 24 h. Exposure of the cornea to APCP was also performed after addition of NAC at a final concentration of 10 mM 1 h prior to APCP treatment, with replenishment in the culture medium every 2 hours. At the chosen time points, corneal samples were snap frozen and stored at -80°C for subsequent use. In a previous study [15], we showed that the helium flow alone did not induce any effect on cell viability and apoptosis of corneal tissues.

### Effects of APCP treatment

The effects of APCP in the absence or presence of 10 mM NAC (HC3-HC4 and HC5-HC6, respectively) compared to unexposed controls (HC1-HC2) were assessed at 6 h post-treatment by histological and immunohistochemical analyses, Western blotting and high-throughput RNA sequencing (RNA-seq). Two additional corneas were subsequently exposed to APCP under the same conditions to measure the expression of selected genes by qPCR at 3, 6 and 24 h post-treatment.

#### Histological and Immunohistochemical analyses

Specimens of APCP-treated or untreated corneas (HC1-HC6) were embedded in OCT (Histo-Line Laboratories, Milano, Italy) and 9 μm cryosections were prepared. Tissue morphology was evaluated by hematoxylin and eosin staining and compared to control tissues.

To assess the expression of 8-oxoguanine DNA glycosylase OGG1 in corneal tissues, cryosections were fixed in 10% formalin for 15 min and processed for immunohistochemistry using a polyclonal rabbit anti-OGG1 (Santa Cruz, Santa Cruz, USA). Briefly, the tissue samples were incubated first with avidin and biotin blocking solutions (Vector Labs, Burlingame, CA, USA) and serum from the same animal species as the secondary antibody (Vector Labs), and then incubated for 60 min with anti-OGG1 at 1:50 in 0.05M Trizma maleate (Sigma, St Louis, MO, USA), pH 7,6. Slides were washed in Trizma maleate, treated with a biotinylated secondary rabbit antibody (Vector Labs) and subsequently treated with alkaline phosphatase ABC kit (Vectstain, Vector Labs). Finally, they were stained with fast red (Sigma) and counterstained with hematoxylin. Negative controls were prepared by omitting the primary antibody.

#### Detection of OGG1 expression by Western Blotting

Corneal tissues exposed to APCP in the presence or absence of 10 mM NAC, as well as the associated control tissues, were maintained in culture for 6 and 24 h post-treatment, washed with ice-cold PBS and snap frozen in liquid nitrogen. Subsequently, cryosections were prepared and treated for 45 min at 4°C with non-denaturing RIPA lysis buffer (1% v/v Triton X-100, 0.5% w/v deoxycholic acid, 10 mM EDTA, 1 μM leupeptin, 150 nM aprotinin, 500 μM 4-(2-aminoethyl) benzenesulfonylfluoride in PBS). After removal of particulate material by centrifugation (15,000 x*g* for 10 min, at 4°C), the supernatants were collected and the protein concentration was determined using the Bio Rad Protein Assay (BioRad, Munich, Germany). About 10 μg of protein lysates were added to the sample loading buffer (62.5 mM Tris pH 6.8, 10% v/v glycerol, 2% w/v sodium dodecyl sulphate, 5% v/v β-mercaptoethanol, and 0.1% w/v bromophenol blue), denaturated at 96°C for 5 min and separated by 8% sodium dodecyl sulphate-polyacrylamide gel electrophoresis (SDS-PAGE). Proteins were transferred to nitrocellulose membrane overnight at 4°C with a constant current of 100 mA, in blotting buffer (25 mM Tris, 192 mM Glycine and 20% Methanol). Non-specific binding sites were blocked by incubating nitrocellulose for 1 h at 22°C in 5% w/v non-fat dry milk in 20 mM TRIS pH 7.6, 150 mM NaCl, 0.1% Tween-20 (TBST). The nitrocellulose membrane was then incubated with polyclonal rabbit anti-OGG1 (Santa Cruz) diluted 1:2000 in TBST for 2 h at room temperature (RT). After three washings of 10 min in TBST nitrocellulose, samples were incubated with peroxidase conjugated anti-rabbit IgG (Thermo Scientific, Rockford, IL, USA), diluted 1:20000 in TBST for 1 h at RT. The antibody reaction was revealed by chemiluminescence using ECL Plus detection reagents (Amersham GE Healthcare, Buckinghamshire, UK). Blots were sequentially incubated at RT for 2 h with monoclonal anti-β-actin antibody (Sigma) diluted 1:4000 and peroxidase conjugated anti-mouse IgG (Amersham) diluted 1:4000 and antidody binding was detected using 3,3’-Diaminobenzidine (Sigma). Densitometric values of OGG1 autoradiographic bands were normalized to corresponding β-actin and expressed as percentage ± SE of the mean control value.

#### Purification of corneal RNA and library preparation

Total RNA was separately purified from two untreated (HC1, HC2) and four APCP-treated corneal samples (HC3-HC6). HC5 and HC6 were treated in the presence of NAC.

Tissues were homogenized using a T-10 Ultra-Turrax (IKA, Staufen, Germany) and total RNA was extracted from each sample with TRIzol (Life Technologies, Carlsbad, CA, USA), according to the manufacturer’s protocol. The RNA concentration of each sample was quantified using the Qubit RN.

A HS Assay Kit (Life Technologies) and RNA quality was assessed with an Agilent Bioanalyzer 2100 (Agilent, Santa Clara, CA, USA). Total RNA was processed for either RNA-seq or qPCR, as indicated below.

Total RNA of samples HC1-HC6 was used to prepare libraries with the TruSeq RNA Sample Preparation kits, per the manufacturer’s instructions (Illumina, San Diego, CA, USA). Each tagged sample was subjected to 100 cycles of sequencing from both ends using one lane of an Illumina Hiseq1000 Sequencer (BMR Genomics, Padova, Italy).

#### Pre-processing, mapping, annotation and analysis of the RNA-seq reads

The 100-bp paired-end reads were extracted using CASAVA (Illumina) and quality was tested using the FastQC suite. For each sequenced sample, reads with a quality score of >Q30 were used to generate a complete FASTQ file, which was then mapped to the human reference genome (build hg19) by the CLC Genomic Workbench v.6 (CLC bio, Aarhus, Denmark) with default parameter settings. After normalization, the resulting aligned reads were analyzed to measure the relative transcript abundance. The expression of each gene was quantified as FPKM (Fragments Per Kilobase of exon per Million fragments mapped, i.e. number of paired-reads mapping to a gene transcript divided by its length in kilobases and by the total number of mapped reads in millions). We used Uniprot gene ontology (GO) annotation to assign gene functions to hg19 IDs. Overall, information on 52,447 unique transcripts was retrieved, with 16,920 of them being assigned to function-defined protein-coding genes, whereas the remaining transcripts referred to putative genes or untranslated RNAs. The Baggerley's test with false discovery rate (FDR) p-value correction was applied to identify genes differentially expressed in the analyzed samples [19]. Fold change (FC) and statistical significance cutoffs were set at 2 and p <0.01, respectively. The hyper-geometric test on annotation was carried out to identify DEG-related GO terms [20]. Raw RNA-seq data were deposited in ENA (accession number PRJEB5765).

#### Quantitative PCR assays

Following primer design with Primer3 [21], the expression levels of 17 selected genes were defined by qPCR to validate the RNA-seq results and evaluate time-related expression trends (Table 2). The specificity of each primer pair was preliminary verified with BLAST searches.

**Table 2:** Gene ID, Ensemble ID and related Forward and Reverse primers used in qPCR.

Gene ID	Ensembl ID	Forward Primer	Reverse Primer
GAPDH	ENSG00000111640	TCAACAGCGACACCCAC	GGGTCTCTCTCTTCCTCTTGTG
ANGPTL7	ENSG00000171819	AGAGATGGAGGACTGGGAGG	TGGTGCTGAAGGCTGTGT
ANKRD1	ENSG00000148677	TGATTATGTATGGCGCGGATCT	GGCTGTCGAATATTGCTTTGGT
ANXA1	ENSG00000135046	ACGCTTTGCTTTCTCTTGCT	TGTCCCCTTTCTCCTTTCTCCT
CASP14	ENSG00000105141	GGGAGAAGATGGGGAGATGG	TGGGATGGTTTGTGGGCT
CCL2*	ENSG00000108691	CAGCAAGTGTCCCAAAGAAGC	AGTGAGTGTTCAAGTCTTCGGA
COL1A1	ENSG00000108821	GGCAAAGATGGACTCAACGG	GGGCAGGAAGCTGAAGTCGAAA
CYP4F11	ENSG00000171903	GTCTACGACCCCTTCCGTTT	ACCACCTTCATCTCAGCCA
DCN*	ENSG00000011465	AGCTCAGGAATTGAAAATGGGG	CGTAAGGGAAGGAGGAAGACC
NOS1	ENSG00000089250	ACATCACCACGCCACCAAC	TCCTCGTACTCCTGCAAACCC
OGG1*	ENSG00000114026	CCAAGAGGTGGCTCAGAAA	GGCGATGTTGTTGTTGGAGGAA
PIM1	ENSG00000137193	GTGCTCCCACACATCAATCC	GTGCTCCCACACATCAATCC
PTX3*	ENSG00000163661	TGTGGGTGGTGGCTTTGATG	CGGATGTGACAAGACTCTGCTC
SERPINE1	ENSG00000106366	CTCTGCCCTCACCAACAT	CGGTCATTCCCAGGTTCTC
SFRP2	ENSG00000145423	GAAATCGGTGCTGTGGCT	CTTGAACTCTCTCTGCCCCT
SOD2*	ENSG00000112096	TTTCAATAAGGAACGGGGACAC	GTGCTCCCACACATCAATCC
SPRR1A*	ENSG00000169474	AATCGCTCCTTTGCACCTC	CCTTCCCCAAATCCATCCTC

GAPDH was used as housekeeping gene, other genes were selected for RNA-seq validation (underlined) or time-related expression analysis (*)

Briefly, 1 μg of total RNA was retro-transcribed into cDNA using oligo(dT)_12–18_, 25 ng primers and 200 U of SuperScript II Reverse Transcriptase (Life Technologies). Quantitative PCR analysis was carried out with the Rotor Gene RG-3000A Real Time PCR system (Corbett Research, Sydney, Australia) using the SYBR Green I dye (Roche, Mannheim, Germany) and combined sense and antisense primers at 300 nM final concentration. We used the following cycling conditions: an initial denaturation step of 10 min at 95°C, 45 cycles of amplification consisting of 15 sec at 95°C, 30 sec at 60°C and 30 sec at 72°C. cDNA samples were analyzed in triplicate. Gene expression was evaluated with ΔCt method using glyceraldehyde-3-phosphate dehydrogenase (GAPDH) as the reference gene. GAPDH was identified also by sequencing data as the most stably expressed housekeeping gene in corneal samples [22]. Results are expressed as gene expression fold change of the treated corneas compared to the control ones.

#### Time-related gene expression changes in the APCP-treated corneas

To evaluate the expression of selected genes, we used two corneal specimens: one was exposed to APCP for 2 min and the other was used as an untreated control. To minimize inter-sample variability of response, both corneas were then cut equally in three pieces, re-incubated with fresh medium and collected (one control and one treated fragment) at 3, 6 and 24 h post-treatment. Following RNA purification, the expression of eight selected genes was evaluated by qPCR in the APCP-treated versus untreated samples for each time point (Table 2).

#### Statistical analysis

Western blotting experiments were performed in duplicate and the data are expressed as mean ± standard error (SEM). Comparisons between groups were made using the Student’s unpaired test. For statistical significance, the assigned P value was p<0.05.

## Results

### APCP did not cause morphological changes of corneal tissue but caused a transient and slight increase in OGG1 protein expression

**Fig 1:**
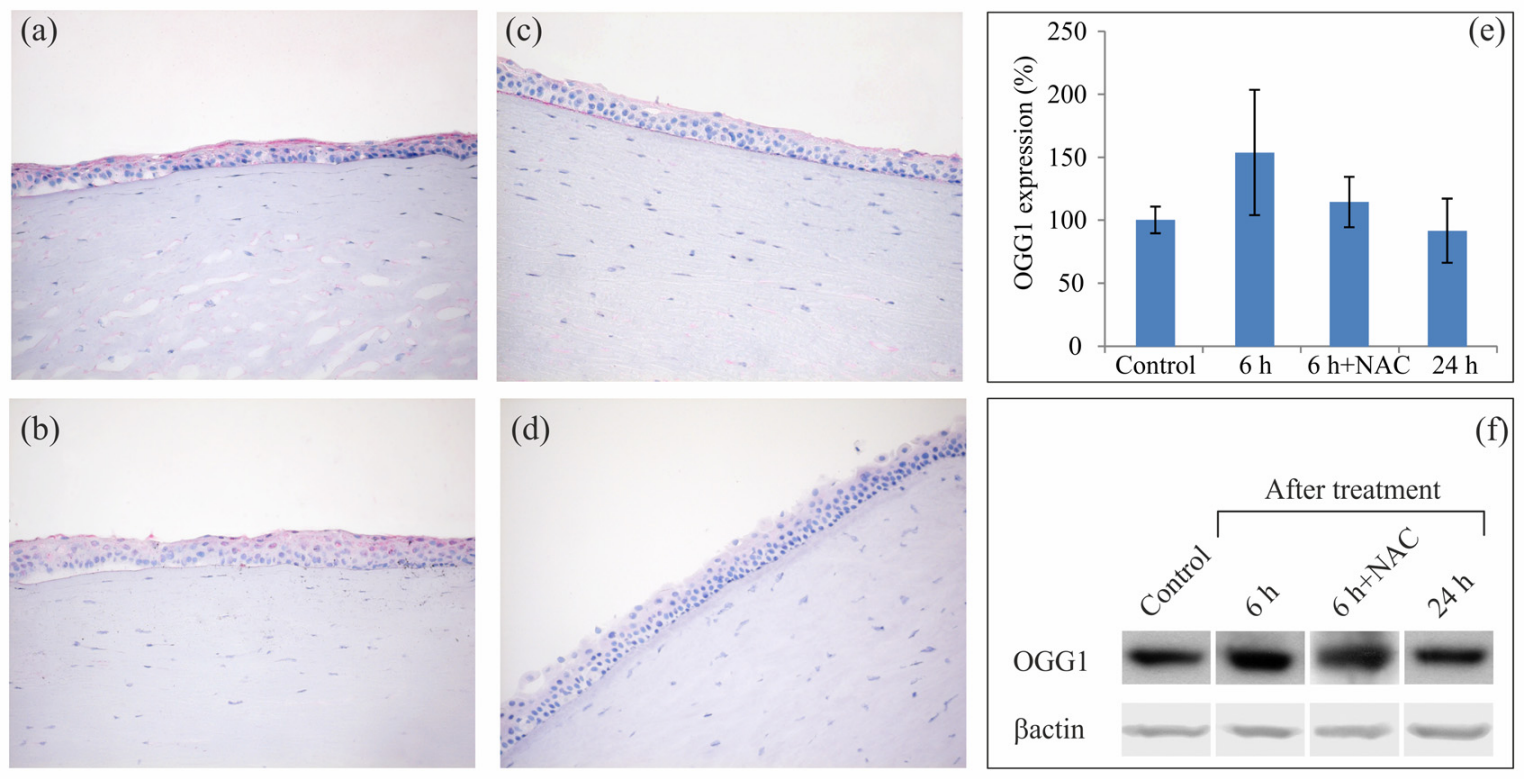
Detection of OGG1 in human corneas treated ex-vivo with APCP. Corneal tissues exposed for 2 min to APCP were analyzed by immunohistochemistry (a-d) and Western Blot (e-f). Frozen sections (5 μm) of corneas treated in the absence (a) or presence (b) of 10 mM NAC were incubated with polyclonal rabbit anti-OGG1 at 6 h post-treatment. Protein immunostaining (in red) was compared to that of untreated controls (c). Negative controls were prepared by omitting the primary antibody (d). For the Western Blot analysis, proteins were extracted at 6 and 24 h post-treatment: the OGG1 protein signal increased at 6 h, and was reduced in the presence of NAC, and returned to values comparable to that of controls within 24 h. Densitometric values of OGG1 autoradiographic bands were normalized to corresponding β-actin and expressed as percentage ± SE of the mean control value.

Irrespective of the addition of 10 mM NAC, *ex vivo* exposure of ex-vivo human corneas to APCP for 2 min did not induce any visible morphological changes when tissues were stained with hematoxylin and eosin (S1 Fig). At 6 hours (h) post-treatment, OGG1 protein was localized by immunohistochemistry in the corneal epithelium, with a slightly more intense signal in APCP-treated corneas than in control samples (Fig 1a–1d). When tissues were exposed to APCP in the presence of NAC, the OGG1 signal was comparable to that of control samples. Western blot analysis demonstrated a slight but not significant increase of OGG1 expression in corneal tissues at 6 h post-treatment, in agreement with previously published data (15), with a return to control levels in 24 h. Notably, at the same sampling time, the NAC addition reduced somewhat the OGG1 signal (Fig 1e and 1f).

### Cornea transcriptome changes at 6 h after exposure to APCP

Next generation sequencing of six human corneal (HC) samples produced nearly 358 million sequencing reads, with 52–67 Mreads/sample and 6 Gbp/sample (Table 1). More than 95% of bases had a quality score of >Q60, indicative of an error every 10^6^ bases. After quality trimming, the average mRNA insert size was 259 bp (StDev 652 bp). About 84–92% of the total reads uniquely aligned to the human genome reference (hg19 release) with 50.3x10^6^ mapped reads/sample on average.

Considering each transcriptome dataset (HC1 to HC6), Principal Component Analysis (PCA) underlined a clear-cut difference between samples HC1-HC2 and HC3-HC6 (S2 Fig). Untreated controls HC1 and HC2 showed very similar gene expression trends, with differences found only in three differentially expressed genes (DEGs), whereas the APCP-treated corneal samples differed by hundreds of DEGs.

**Fig 2:**
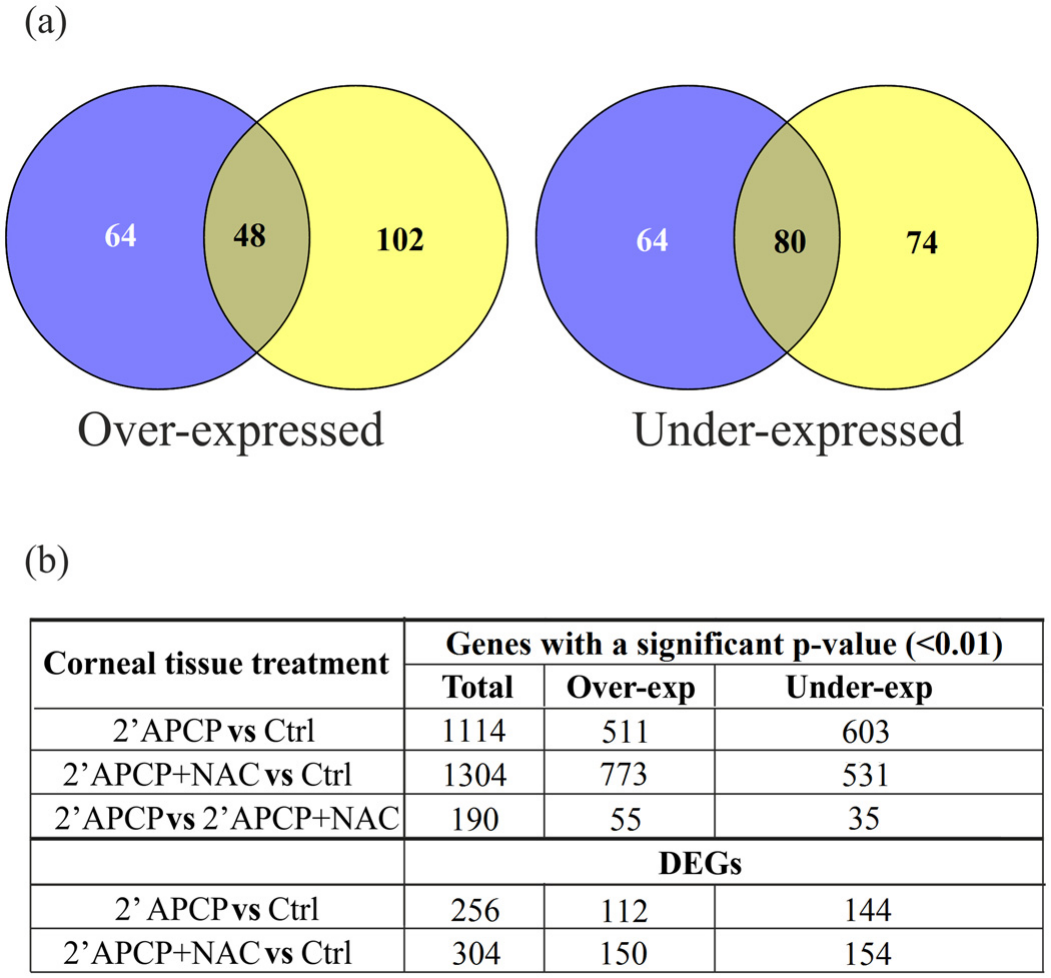
Differentially expressed corneal genes (DEGs) at 6 h after exposure to APCP. (a) over- and under-expressed DEGs are shown as common (overlapping area) or exclusive to the APCP (left) or APCP+NAC (right) treatments; (b) number of total (Baggerly’s test FDR p-value <0.01) and DEG, over- and under-expressed genes detected in HC1-HC6 samples, paired per condition.

To characterize the transcriptome of corneas exposed to APCP with or without NAC, we subsequently analyzed significant gene expression changes in the HC1-HC6 datasets, pairing the biological replicates per condition, and identifying 2608 genes with an FDR-corrected p-value lower than 0.01 (the complete lists of genes are reported in S1 Dataset). Among these genes, we identified 256 DEGs (112 over- and 144 under-expressed) in APCP-treated corneas and 304 DEGs (150 over- and 154 under-expressed) in APCP+NAC-treated corneas compared to control corneas (Fig 2). Computing the number of common and exclusive DEGs in the APCP-treated corneas vs. the untreated controls, we found a consistent fraction of NAC-exclusive over-expressed (48%) and under-expressed (34%) DEGs.

**Fig 3:**
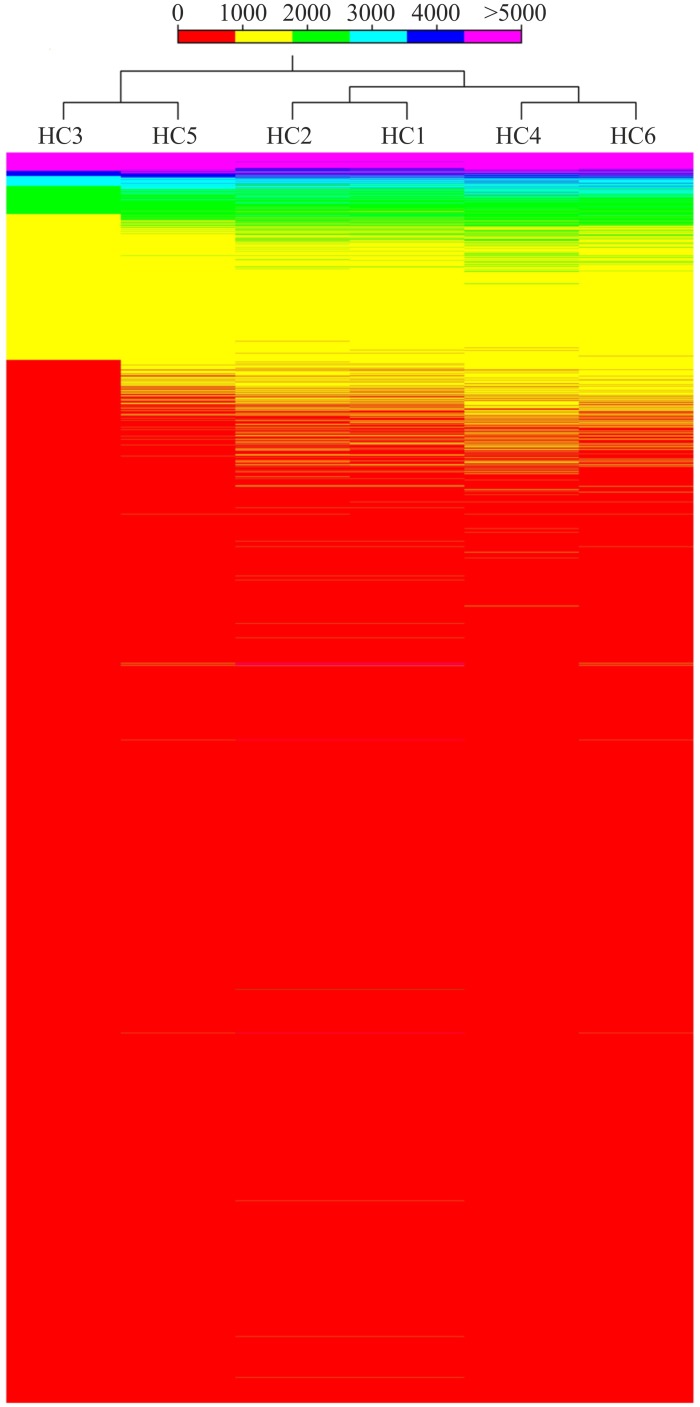
Hierarchical clustering performed on the whole normalized dataset. 52,447 genes, Manhattan correlation, complete linkage.

Hierarchical clustering of the HC1-HC6 subsets (total modulated genes) essentially discriminated the controls (1–2) from the other APCP-treated samples and all six samples correctly clustered when DEGs altered more than 2-fold were considered (Fig 3). The hyper-geometric test on annotation extracted the GO categories from the subsets of over- and under-expressed genes by comparing them with the whole expression dataset (S2 Dataset). In the samples treated with APCP or APCP+NAC relative to the untreated controls, 231/511 (45.2%) and 294/773 (38%) modulated genes (p<0.01) concerned regulation or regulatory processes, respectively (154 and 181 genes were specifically related to signaling and transduction). In the presence of NAC, the amount of genes related to transcription or RNA increased from 89 (17.4%) to 158 (20%), and those involved in protein translation from 23 (4.5%) to 63 (8%). Apoptosis-related genes were observed in similar proportions, i.e. 63 (12.3%) and 86 (11.7%), respectively. Under the same conditions (APCP; APCP+NAC), also genes related to ubiquitin/ubiquitination (31; 27), inflammation/inflammatory processes (22; 22), innate responses (18; 16), and oxidation/oxidative processes (8; 10) were somewhat up-regulated. Genes associated with damage and repair (16; 24) and positive regulation of DNA damage response (4; 5) were also up-regulated to some degree (<2 fold). Among them, we detected the serine/threonine-protein kinase ATR (1.66x with NAC), apoptosis-stimulating TP53-Binding Protein 2 (1.39x), UV excision repair RAD23 homolog B (1.19x) and a DNA damage-inducible transcript 3 protein DDIT3 (1.19x). A similar finding regarded genes involved in cell cycle and chromatin accessibility, such as the cyclin-dependent kinases CDKN2AIP (1.57x; 1.47x with NAC), CDKN1A (1.35x), CDK7 (1.21x with NAC), ADP-ribosylation factor-like protein 8B (1.3x) and PARP10 (1.58x; -1.29x with NAC).

**Fig 4:**
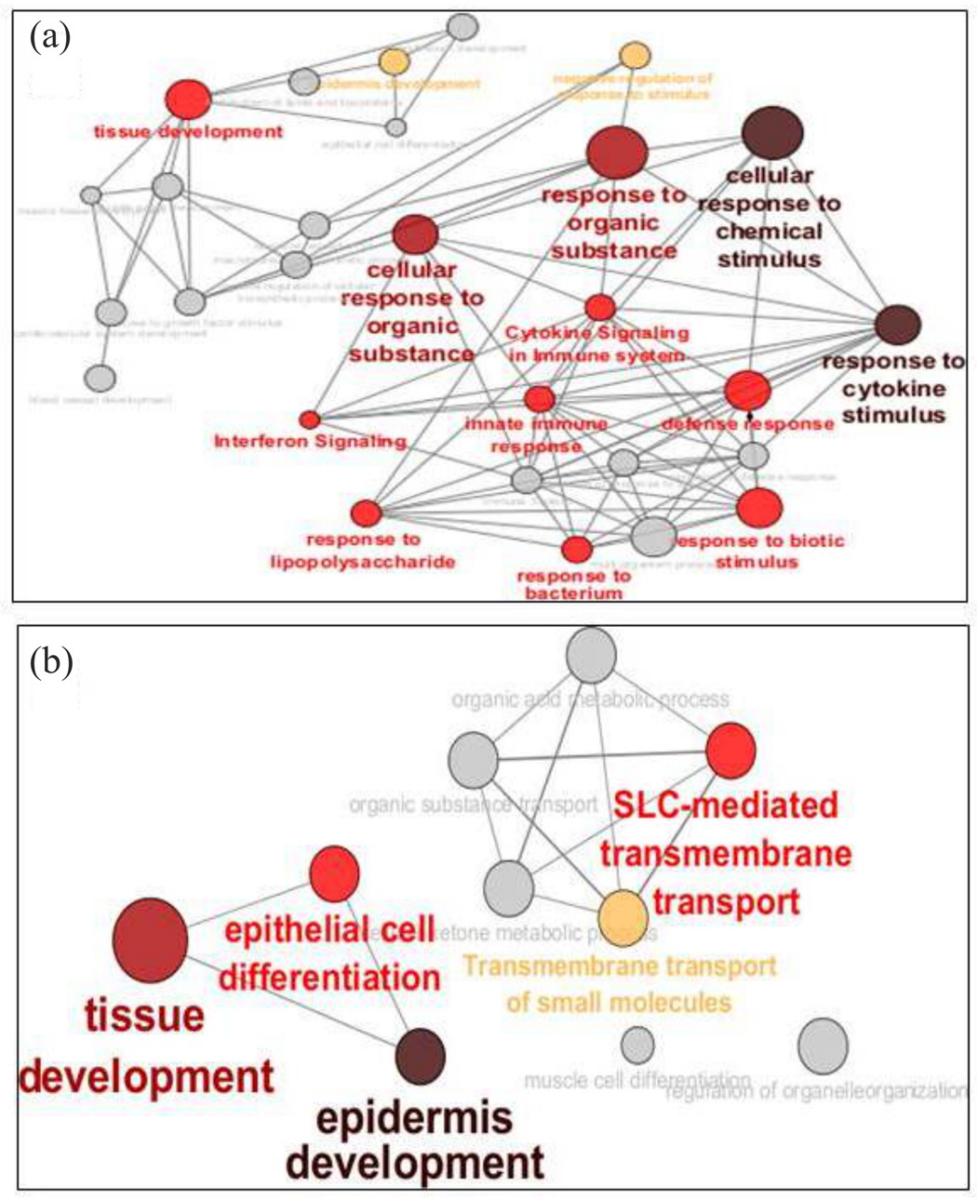
Gene Ontologies most represented in the corneal genes up-regulated by APCP. Gene Ontologies most represented in the corneal genes up-regulated by APCP in the absence (a) or presence (b) of NAC. Size and gray scale color of the circles reflect the importance of cell pathways, represented as functionally connected nodes.

Fig 4 provides a snapshot of the most represented GO categories in all up-regulated genes. Based on the whole set of expression trends, the corneal cells reacted to APCP by activating various pathways of response, including those triggering innate defenses, whereas the reaction to APCP in the presence of NAC enhanced pathways related to tissue development, transport processes, cell differentiation and metabolism.

After APCP treatment, many DEGs showed common expression trends independent of NAC. Among the most over-expressed genes, we found transcripts for glutathione S-transferase Mu, beta defensins 103, survival motor neuron protein, nicotinamide phosphoribosyltransferase, small proline-rich protein 2F, annexin A8 (ANXA8) and CYP4F11. Many transcripts encoding Ig- protein regions and granzyme H were among the most under-expressed (S1 Dataset).

### Quantitative real time PCR-validated selected transcriptome data

**Fig 5:**
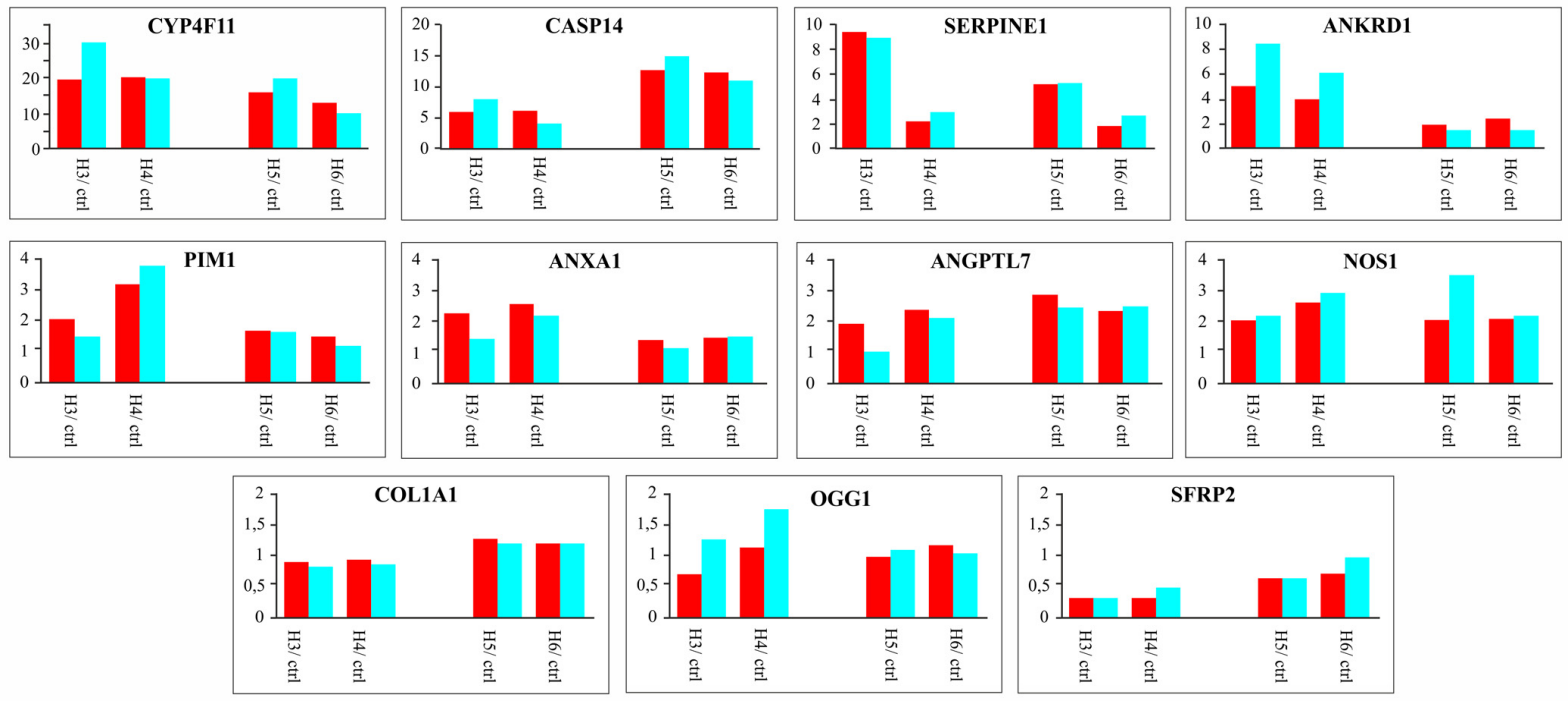
Expression levels of selected genes as measured by RNAseq and qPCR. The expression values generated by RNA-seq (red) or qPCR (blu) for eleven selected genes using the same corneal samples (Table 1) are compared. Values detected for each gene were normalized to GAPDH expression and reported as a ratio between APCP-exposed samples and unexposed controls.

We examined by qPCR the expression of eleven genes involved in cell responses to oxidative stress, DNA repair and regulation of apoptotic cell death (Table 3). We normalized their expression levels to the housekeeping gene GAPDH in both the RNA-seq and qPCR datasets, and calculated the ratio between each APCP-treated sample and averaged controls. Direct data comparison indicated that qPCR was less sensitive than RNA-seq analysis; however, the two datasets revealed considerable similarity when the ratio between treated and control samples was used (Fig 5)

**Table 3:** A selection of corneal genes to this study.

		2' APCP		2' APCP + NAC	
*Gene ID*	*Gene description*	*FC*	*Rank*	*FC*	*Rank*
ANGPTL7^	angiopoietin-like 7	2.08	619	2.63	541
ANKRD1^	ankyrin repeat domain 1 (cardiac muscle)	4.28	5050	2.13	7870
ANXA1*^	annexin A1	24.73	92	21.21	139
ATR	serine/threonine-protein kinase ATR	-	9809	1.66	9164
CASP14*^	caspase 14, apoptosis-related cysteine peptidase	5.84	9588	12.55	6632
CCL2*	chemokine (C-C motif) ligand 2	-	115	-	169
COL1A1^	collagen, type I, alpha 1	-	5269	-	3946
DCN*	decorin	-	113	-	115
DDIT3	D0 damage-inducible transcript 3 protein	-	1878	1.49	2031
DEFB4A	Beta-defensin 4A	-	1276	9.42	1175
NAMPT	nicotinamide phosphoribosyltransferase	113.53	503	104.01	741
NOS1^	nitric oxide synthase 1 (neuro0l)	-	11600	-	11883
OGG1*^	8-oxoguanine D0 glycosylase	-	9855	-	9242
PIM1^	pim-1 oncogene	-	2019	-	1229
PTX3*	pentraxin 3, long	-	215	-	649
RAD23B	UV excision repair protein RAD23 homolog B	1.19	838	-	808
SERPINE^	serpin peptidase inhibitor	-	689	-	1162
SFRP2^	secreted frizzled-related protein 2	-	12558	-	10418
SMN2	Survival motor neuron protein	145.99	4017	163.46	3609
SOD2*	superoxide dismutase 2, mitochondrial	-	105	-	161
SPRR1A*	Cornifin-A	2.62	138	1.6	227
SPRR2F	small proline-rich protein 2F	35.07	9347	-	9521
TP53BP2	Apoptosis-stimulating of p53 protein 2	1.39	5952	-	6795

A selection of DEGs for at least one treatment or genes selected for RNA-seq validation (^) or time-related expression analysis (*). Where genes are DEG, fold change value and absolute ranks in the transcriptome was reported. FC, Fold Change.

### Time-related expression changes of selected genes in APCP-treated corneas

We investigated the expression of eight genes up-regulated after exposure to cold plasma (OGG1, ANXA1, CASP14, SOD2, PTX3, DCN, SPRR1A, and CCL2; Table 3) using two corneas (one exposed to APCP, one untreated control). Aiming to minimize the inter-individual variability of response, we divided each twin cornea into three pieces and collected one by one at 3, 6 and 24 h post-treatment for qPCR analysis. At 3 and 6 h post-treatment, APCP increased at different degree the expression of all selected genes, namely SPRR1A, SOD2, CASP14, ANXA1 and OGG1 (only the PTX3 expression level was less than 1 at 6 h post-treatment).

**Fig 6:**
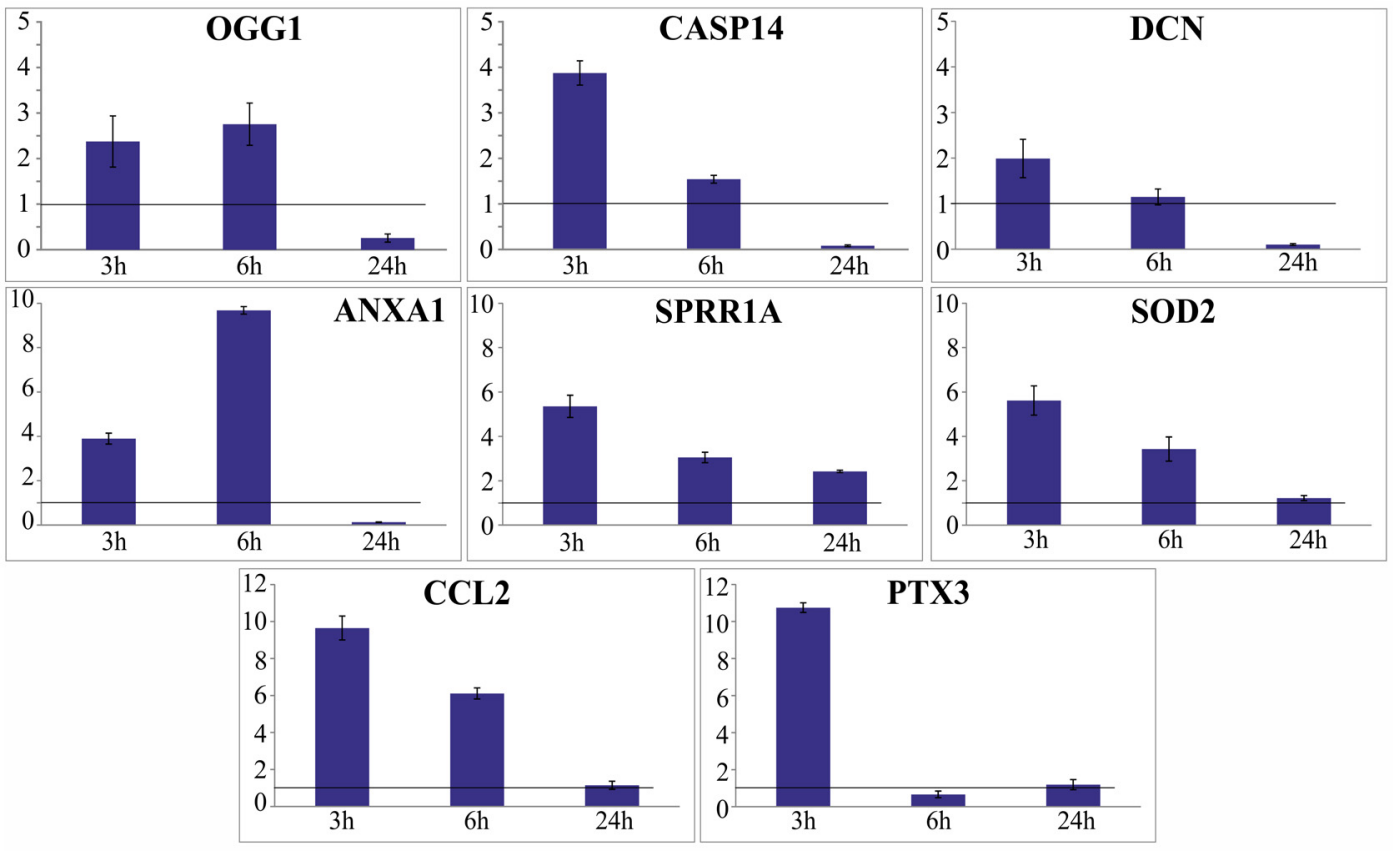
qPCR analysis of selected genes at different time points. Two human corneas were used to evaluate the expression of specific genes by qPCR at selected time points from the exposure to APCP. To minimize the variability of response, both corneas, one used as negative control and the other exposed to 2 min APCP, were divided into three pieces, then collected at 3, 6 and 24 h post-treatment. Expression levels of the target genes, detected in the treated corneal sample relative to the untreated sample were normalized to GAPDH levels.

The expression of all genes returned to background levels after 24 h, with the exception of SPRR1A (Fig 6).

## Discussion

A cold plasma is a partially ionized gas, typically produced by the application of a steady or time-varying voltage. The power level is kept low enough that only electrons are heated to high temperatures (of the order of 10,000 K), whereas the ions and the neutral gas are at or near the atmospheric pressure (hence the “cold” attribute). While ionization is easier at low pressure, cold plasmas used in biomedical applications are produced at atmospheric pressure. The effect of cold plasma on micro-organisms and eukaryotic cells depends on the type of gas and device used to produce it [23]. As previously mentioned, a two-minute application of the APCP was effective in killing ocular pathogens. However, this dose of APCP induced a transitory formation of ROS, 8-OHdG, and increased expression of OGG1 protein in ocular cells and tissues [15]. Moreover, in a previous study we found that fibroblast proliferation was ROS-dependent since incubation of cells with an antioxidant agents such NAC, significantly reduced the biological effects [16].

In the present study, transcriptome sequencing, qPCR and Western Blot analyses performed on human corneas at 6 h post-treatment confirmed a mild and transient increase of OGG1 transcripts and proteins, which was slightly reduced by the antioxidant NAC. Furthermore, irrespective of the addition of NAC, the levels of OGG1 mRNA and protein decreased to control levels at 24 h post-treatment. Although differences among corneal specimens may reflect individual responses to the stimulus, the experimental trends observed in this study confirm some of the previous results obtained on *ex-vivo* cornea specimens [15].

The intensity of APCP-induced oxidative stress has been ascribed to short-lived chemical species such as hydrogen peroxide and hydroperoxy radicals [24–25]. If not adequately counterbalanced, propagation of oxidative reactions may impair cell physiology, whereas clusters of oxidative DNA lesions inefficiently repaired could contribute to mutations and degenerative processes [26–27].

The different response of prokaryotic vs. eukaryotic cells to APCP is likely due to the haploid condition and differences in cell structure and DNA repair pathways. In fact, DNA mutations are immediately evident in the phenotype of haploid cells and, when occurring in essential genes, can lead to bacterial death also because of the speed of transcription and protein synthesis.

The effects of cold plasma on eukaryotic cells and tissues are certainly dependent on gas flow, distance from the plasma source and exposure time [28–29]. In our study we compared the effect of APCP on corneal tissue in presence or absence of NAC to evaluate how this antioxidant compound modulates the response of human corneal cells to APCP. At 6 h from exposure to APCP, we detected similar amounts of over- and under-expressed genes (the number of over-expressed genes prevailed in the presence of NAC). In corneas exposed to APCP for 2 min and sampled at 6 h post-treatment, the transcriptome analysis revealed only a slight up-regulation of a number of genes involved in sensing and repair of DNA damage. The considerable up-regulation of genes related to the response to stimuli, signal transduction and RNA processes suggested a broad-ranging cell activity, reminiscent of hormetic responses paradoxically observed at low doses of ionizing radiations or toxic chemicals [30–31].

In the treated corneas, defensin transcripts were the most abundant (over-expressed) regardless of NAC addition. We also found up-regulated transcripts denoting interleukin-associated proteins, interferon-related (e.g. GBP2, ISG20) and cell defense proteins such as cytochrome CYP4F11 (19.25x; 14.86x with NAC) and glutathione peroxidase (3.14x; 4.98x with NAC). These facts, suggest a reinforcement of innate protective mechanisms even in *ex vivo* conditions.

Cellular proteins are highly sensitive to oxidative stress and recent studies demonstrated that APCP causes protein unfolding in aqueous solution [24] and accumulation of non-functional proteins in bacteria exposed to plasma [32]. At 6 h post-treatment and irrespective of NAC addition, the plasma exerted very slight up-regulation (from 1.1 to 1.4 fold) of chaperon proteins and genes related to ubiquitination (UBA1, UBA2, UBE2D3, UBE2H, UBE2D2) in corneal tissues, indicating that a 2-min APCP does not cause relevant protein degradation. These data are consistent with other recent data pointing to a stimulatory effect of ROS on liver and intestinal fibroblast-like primary cells [16].

It has also been reported that human cells can enter apoptosis in a treatment- and time-dependent manner after exposure to cold plasma [13,25]. The TUNEL assay previously performed on corneal cells and tissues treated with APCP for 2 min did not reveal significant apoptotic effects. Transcriptome analysis performed on treated corneas indicated up-regulation of ANXA8 (24.73x, 21.21x with NAC) and ANXA1 (2.32x, only 1.46x with NAC), but not ANXA5. Annexin A1 is involved in anti-inflammatory signaling, kinase regulation, apoptosis and differentiation. The considerable basal and APCP-induced levels of ANXA1 transcripts support its role in efficient ROS scavenging [33–34]. qPCR analyses confirmed an over-expression of ANXA1 at 3 and 6 h after exposure to APCP. The functional role of ANXA8 is not completely understood but is certainly associated with terminal differentiation of epithelial cells [35]. In addition, the anti-apoptotic protein CASP14, which has been reported in cornifying epithelia [36], was clearly over-expressed (5.84x; 12.85x with NAC). Other genes related to keratinization, like cornifelin (CNFN), cornifin- A (SPRR1A), small proline rich protein (SPRR) 2D and SPRR2E were also found to be over-expressed. Up-regulation of SPRR1A was confirmed by qPCR analysis at different time points (6x after 3 h; 3x after 6 h). Several studies have suggested that SPRRs are also related to increased epithelial proliferation and differentiation [37]. However, their role in pathophysiological processes requires further study.

In conclusion, a new antimicrobial therapy based on cold plasma needs to have a proven safety profile. The utilization of cold plasma in medicine is well described, in particular in dermatology where the benefit of safe skin disinfection and improved healing has been demonstrated [38,12]. Our previous studies proved the effectiveness of disinfection of ocular surfaces with cold plasma. In the present study, we confirm a transient and moderate oxidative damage in corneal tissues exposed to APCP for 2 min. The overall gene expression changes reveal a stimulatory or protective response rather than a weakness of cell functioning. In the presence of the antioxidant NAC, which was previously shown to reduce the burst of intracellular ROS, the number of over-expressed genes was higher, suggesting a manifold cell response to the APCP exposure. Moreover, the expression trends of one DNA repair protein (OGG1) and the over-expression of an anti-apoptotic CASP14 transcript in the presence of NAC support its protective role against potential ROS-induced damage induced by APCP treatment. Further studies will adequately assess the real influence of this molecule in APCP-treated tissues *in vitro* and *in vivo*

## Supporting Information

S1 Dataset All genes displaying a statistically significant p-value (Baggerly’s test with FDR corrected p-value cutoff <0.01) are listed as over-expressed (A) or under-expressed (B) with gene ID, fold change, FDR corrected p value, GO biological process, gene name and detected expression values (three experimental conditions in paired comparisons). Genes displaying also a fold change higher than 2 (see M&M) are considered as ‘DEG’ and are highlighted in grey.
(XLSX)

S2 Dataset Hyper-Geometric Test on Annotation performed on over- and under-DEGs. ID and description of the resulting GO categories, number of grouped genes and associated p-value.
(XLSX)

S1 Fig Morphology of human corneas untreated (a) or treated ex-vivo with APCP in the absence (b) or presence (c) of NAC. Tissue morphology at 6 h from exposure to APCP (5 μm sections, hematoxylin and eosin, 100x magnification).
(TIF)

S2 Fig Principal Component Analysis (PCA) performed on HC1-HC6 corneal transcriptomes. (TIF)

## References

[1] Garreis F, Gottschalt M, Paulsen FP (2010) Antimicrobial peptides as a major part of the innate immune defense at the ocular surface. Dev Ophthalmol. 45:16–22. 10.1159/000315016 20502023

[2] Thomas PA, Geraldine P (2007) Infectious keratitis. Curr Opin Infect Dis. 20(2):129–41. 17496570 10.1097/QCO.0b013e328017f878

[3] Del Monte DW, Kim T (2011) Anatomy and physiology of the cornea. J Cataract Refract Surg. 37 (3): 588–98. 10.1016/j.jcrs.2010.12.037 21333881

[4] Knickelbein JE, Buela KA, Hendricks RL (2014) Antigen-presenting cells are stratified within normal human corneas and are rapidly mobilized during ex vivo viral infection. Invest Ophthalmol Vis Sci. 55(2):1118–23. 10.1167/iovs.13-13523 24508792 PMC3934539

[5] McDonald EM, Ram FS, Patel DV, McGhee CN (2014) Topical antibiotics for the management of bacterial keratitis: an evidence-based review of high quality randomised controlled trials. Br J Ophthalmol. 98(11):1470–7. 10.1136/bjophthalmol-2013-304660 24729078

[6] Fridman G, Friedman G, Gutsol A, Shekhter AB, Vasilets VN, Fridman A, et al (2008) Applied Plasma Medicine. Plasma Processes Polym. 5(6):503–533.

[7] Kong MG, Kroesen G, Morfill G, Nosenko T, Shimizu T, van Dijk J, et al (2009) Plasma medicine: an introductory review New J Phys. 11:115012.

[8] Ermolaeva SA, Varfolomeev AF, Chernukha MY, Yurov DS, Vasiliev MM, Kaminskaya AA, et al (2011) Bactericidal effects of non-thermal argon plasma in vitro, in biofilms and in the animal model of infected wounds. J Med Microbiol. 60(Pt 1):75–83. 10.1099/jmm.0.020263-0 20829396

[9] Ehlbeck J, Schnabel U, Polak M, Winter J, von Woedtke T, Brandenburg R, et al (2011) Low temperature atmospheric pressure plasma sources for microbial decontamination. J Phys D Appl Phys. 44: 013002.

[10] von Woedtke Th, Metelmann HR, Weltmann KD (2014) Clinical Plasma Medicine: State and Perspectives of in Vivo Application of Cold Atmospheric Plasma. Contrib Plasm Phys. 54(2):104–117.

[11] Alekseev O, Donovan K, Limonnik V, Azizkhan- Clifford J (2014) Nonthermal dielectric barrier discharge (DBD) plasma suppresses Herpes Simplex Virus Type 1 (HSV-1) replication in corneal epithelium. Transl Vis Sci Technol. 3(2):2 24757592 10.1167/tvst.3.2.2 PMC3969218

[12] Brehmer F, Haenssle HA, Daeschlein G, Ahmed R, Pfeiffer S (2014) Alleviation of chronic venous leg ulcers with a hand-held dielectric barrier discharge plasma generator (PlasmaDerm VU-2010): results of a monocentric, two-armed, open, prospective, randomized and controlled trial (NCT01415622). J Eur Acad Dermatol Venereol. 29(1):148–55. 10.1111/jdv.12490 24666170

[13] Kalghatgi S, Kelly CM, Cerchar E, Torabi B, Alekseev O, Fridman A, et al (2011) Effects of non-thermal plasma on mammalian cells. PLoS One 6(1):e16270 10.1371/journal.pone.0016270 21283714 PMC3025030

[14] Graves DB (2012) The emerging role of reactive oxygen and nitrogen species in redox biology and some implications for plasma applications to medicine and biology. J Phys D Appl Phys 45:263001.

[15] Brun P, Brun P, Vono M, Venier P, Tarricone E, Deligianni V, et al (2012) Disinfection of ocular cells and tissues by atmospheric-pressure cold plasma. PLoS One 7(3):e33245 10.1371/journal.pone.0033245 22432007 PMC3303808

[16] Brun P, Pathak S, Castagliuolo I, Palù G, Brun P, Zuin M, et al (2014) Helium generated cold plasma finely regulates activation of human fibroblast-like primary cells. PLoS One 9(8):e104397 10.1371/journal.pone.0104397 25127477 PMC4134215

[17] Dobrynin D, Friedman G, Fridman A, Starikovskiy A (2011) Inactivation of bacteria using dc corona discharge: role of ions and humidity. New J Phys. 13:103033 22403515 10.1088/1367-2630/13/10/103033 PMC3295596

[18] Martines E, Zuin M, Cavazzana R, Gazza E, Serianni G, Spagnolo S, et al (2009) A novel plasma source for sterilization of living tissues. New J Phys. 11:115014.

[19] Baggerly KA, Deng L, Morris JS, Aldaz CM (2003) Differential expression in SAGE: accounting for normal between-library variation. Bioinformatics 19(12):1477–83. 12912827 10.1093/bioinformatics/btg173

[20] Tian X, Joo J, Zheng G, Lin JP (2005) Robust trend tests for genetic association in case-control studies using family data. BMC Genet. 6(Suppl 1):S107 16451563 10.1186/1471-2156-6-S1-S107 PMC1866832

[21] Rozen S, Skaletsky H (2000) Primer3 on the WWW for general users and for biologist programmers. Methods Mol Biol. 132:365–86. 10547847 10.1385/1-59259-192-2:365

[22] Kulkarni B, Mohammed I, Hopkinson A, Dua HS (2011) Validation of endogenous control genes for gene expression studies on human ocular surface epithelium. PLoS One 6(8):e22301 10.1371/journal.pone.0022301 21857920 PMC3152287

[23] Isbary G, Shimizu T, Li YF, Stolz W, Thomas HM, Morfill GE, et al (2013) Cold atmospheric plasma devices for medical issues. Expert Rev Med Devices 10:367–77 10.1586/erd.13.4 23668708

[24] Takai E,. Kitano K, Kuwabara J, Shiraki K (2012) Protein Inactivation by Low-temperature Atmospheric Pressure Plasma in Aqueous Solution. Plasma Process Polym. 9:77–82.

[25] Bekeschus S, Kolata J, Winterbourn C, Kramer A, Turner R, Weltmann KD, et al (2014) Hydrogen peroxide: a central player in physical plasma-induced oxidative stress in human blood cells. Free Radic Res 48(5):542–9. 10.3109/10715762.2014.892937 24528134

[26] Delaney S, Jarem DA, Volle CB, Yennie CJ (2012) Chemical and biological consequences of oxidatively damaged guanine in DNA. Free Radical Res. 46(4):420–441. 22239655 10.3109/10715762.2011.653968 PMC3646368

[27] Winczura A, Zdżalik D, Tudek B (2012) Damage of DNA and proteins by major lipid peroxidation products in genome stability. Free Radical Res. 46(4):442–459. 22257221 10.3109/10715762.2012.658516

[28] Ahn HJ, Kim KI, Kim G, Moon E, Yang SS, Lee JS (2011) Atmospheric-Pressure Plasma Jet Induces Apoptosis Involving Mitochondria via Generation of Free Radicals. PLoS One 6(11):e28154 10.1371/journal.pone.0028154 22140530 PMC3226649

[29] Vandamme M, Robert E, Lerondel S, Sarron V, Ries D, Dozias S, et al (2012). ROS implication in a new antitumor strategy based on non-thermal plasma. Int J Cancer 130(9):2185–2194. 10.1002/ijc.26252 21702038

[30] Calabrese EJ, Mattson MP (2011) Hormesis provides a generalized quantitative estimate of biological plasticity. J Cell Commun Signal. 5(1):25–38. 10.1007/s12079-011-0119-1 21484586 PMC3058190

[31] Calabrese EJ. (2013) Biphasic dose responses in biology, toxicology and medicine: accounting for their generalizability and quantitative features. Environ Pollut. 182:452–60. 10.1016/j.envpol.2013.07.046 23992683

[32] Lackmann JW, Schneider S, Edengeise E, Jearzina F, Brinckmann S, Steinborn E, et al (2013) Photons and particles emitted from cold atmospheric-pressure plasma inactivate bacteria and biomolecules independently and synergistically. J R Soc Interface 10(89):20130591 10.1098/rsif.2013.0591 24068175 PMC3808546

[33] Madureira PA, Waisman DM. (2013) Annexin A2: the importance of being redox sensitive. Int J Mol Sci. 14:3568–94. 10.3390/ijms14023568 23434659 PMC3588059

[34] Hoque M, Rentero C, Cairns R, Tebar F, Enrich C, Grewal T (2014) Annexins—Scaffolds modulating PKC localization and signaling. Cell Signal. 26(6):1213–1225. 10.1016/j.cellsig.2014.02.012 24582587

[35] Runkel F, Michels M, Franken S, Franz T (2006) Specific expression of annexin A8 in adult murine stratified epithelia. J Mol Histol. 37(8–9):353–9. 17082908 10.1007/s10735-006-9063-4

[36] Denecker G, Ovaere P, Vandenabeele P, Declercq W (2008) Caspase-14 reveals its secrets. J Cell Biol. 180:451–8. 10.1083/jcb.200709098 18250198 PMC2234247

[37] Carregaro F, Stefanini AC, Henrique T, Tajara EH (2013) Study of small proline-rich proteins (SPRRs) in health and disease: a review of the literature Arch Dermatol Res. 305:857–66. 10.1007/s00403-013-1415-9 24085571

[38] Isbary G, Heinlin J, Shimizu T, Zimmermann JL, Morfill G, Schmidt HU, et al (2012) Successful and safe use of 2 min cold atmospheric argon plasma in chronic wounds: results of a randomized controlled trial Br J Dermatol. 167(2):404–10. 10.1111/j.1365-2133.2012.10923.x 22385038 PMC7161860

