# Supplementary figures and images for: Atmospheric-Pressure Cold Plasma Induces Transcriptional Changes in Ex Vivo Human Corneas
Source: PLoS One. 2015 Jul 23;10(7):e0133173. doi: 10.1371/journal.pone.0133173 (PMC4512711; doi:10.1371/journal.pone.0133173)

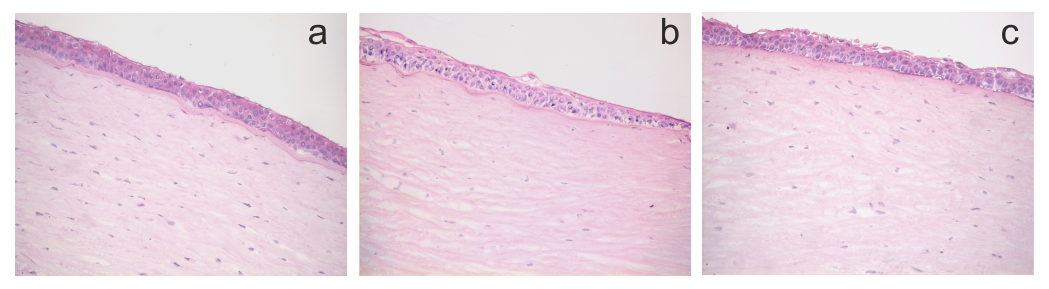

Supplement: S1 Fig — Tissue morphology at 6 h from exposure to APCP (5 μm sections, hematoxylin and eosin, 100x magnification). (TIF) [file pone.0133173.s003.tif]

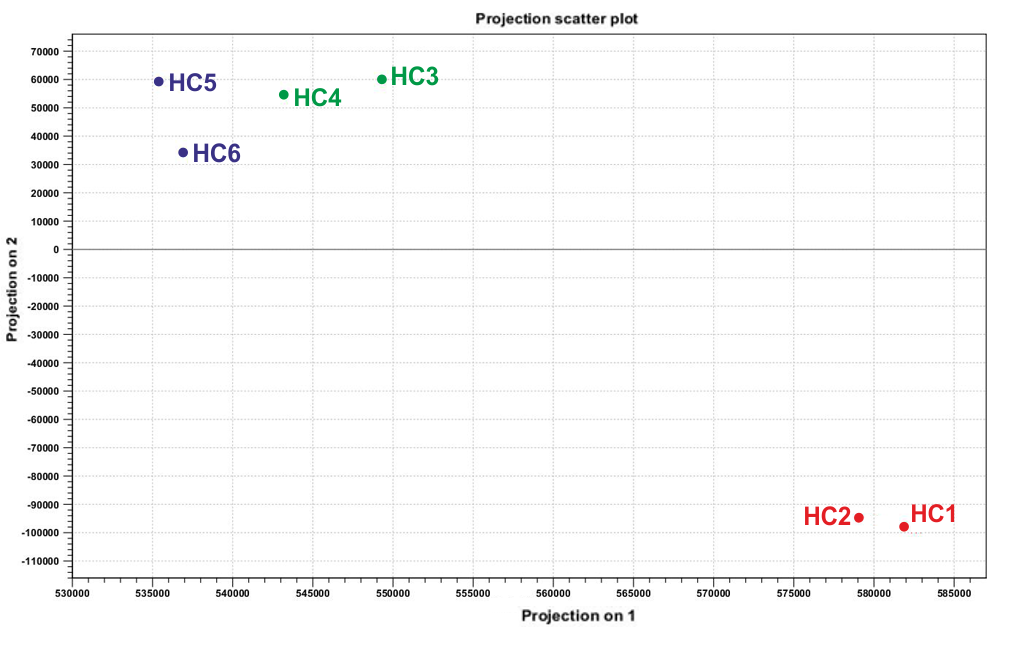

Supplement: S2 Fig — (TIF) [file pone.0133173.s004.tif]
